# Overexpression of LGR-5 as a Predictor of Poor Outcome in Patients with Hepatocellular Carcinoma

**DOI:** 10.3390/ijerph16101836

**Published:** 2019-05-23

**Authors:** Chih-Jan Ko, Chia-Jung Li, Meng-Yu Wu, Pei-Yi Chu

**Affiliations:** 1 Department of General Surgery, Changhua Christian Hospital, Changhua 500, Taiwan; 91681@cch.org.tw; 2 School of Medicine, Kaohsiung Medical University, Kaohsiung 807, Taiwan; 3 Department of Obstetrics and Gynecology, Kaohsiung Veterans General Hospital, Kaohsiung 813, Taiwan; nigel6761@gmail.com; 4 Department of Emergency Medicine, Taipei Tzu Chi Hospital, Buddhist Tzu Chi Medical Foundation, New Taipei 231, Taiwan; skyshangrila@gmail.com; 5 Department of Emergency Medicine, School of Medicine, Tzu Chi University, Hualien 970, Taiwan; 6 School of Medicine, College of Medicine, Fu Jen Catholic University, New Taipei 242, Taiwan; 7 Department of Pathology, Show Chwan Memorial Hospital, Changhua 500, Taiwan; 8 Department of Health Food, Chung Chou University of Science and Technology, Changhua 510, Taiwan

**Keywords:** hepatocellular carcinoma, LGR-5, immunohistochemical markers, outcome

## Abstract

Hepatocarcinogenesis and distant metastasis pose major challenges for physicians. They are regulated by several genes, such as *AKT*, *JUK*, *Wnt*, and *P53*, and their expression activates several important processes such as cell proliferation, migration, motility, and interaction in the microenvironment. The leucine-rich repeat-containing G-protein-coupled receptor 5 (LGR-5) is a novel biomarker, particularly in stem cells, and is involved in embryogenesis, tumor development, and tumor cell signal transduction. Here, we investigated LGR-5 expression using immunohistochemistry and analyzed the correlation between clinical features and prognosis in patients with hepatocellular carcinoma (HCC). We found that LGR-5 expression was higher in tumor tissues than in normal liver tissues, and that high LGR-5 expression possibly favored poor outcomes in HCC, especially in well/moderate differentiation grade, hepatitis C virus (HCV)-negative, and hepatitis B virus (HBV)-positive groups. Thus, the LGR-5 marker is suggested to be a routine biomarker for poor prognosis, thereby providing a platform for anti-LGR-5-targeted therapy in the future.

## 1. Introduction

Hepatocellular carcinoma (HCC) is a major cause of cancer-related deaths with increasing incidence and high mortality rates (35.5/100,000). Several risk factors, such as hepatitis B virus (HBV), hepatitis C virus (HCV), alcoholic liver disease, and non-alcoholic fatty liver disease, have been reported as causative factors in previous studies [1,2,3]. Chronic inflammation leading to cell death and regeneration has been observed, forming a vicious cycle in the promotion of hepatocarcinogenesis [4]. The tumor cells activate the release of growth factors and cytokines, thereby causing tumor progression and distant metastasis. Unfortunately, current therapeutic strategies are facing several challenges such as tumor resistance and host cell toxicity [4]. Thus, a highly sensitive and specific prediction assay utilizing a new target is necessary for the early prediction of HCC. 

In recent studies, the available treatments faced two major challenges, namely carcinogenesis and distant metastases, both of which impaired patient outcomes. A previous study reported that high-grade histology and distant metastases favored advanced and invasive tumor progression with poor clinical outcomes in HCC patients [5]. Several genes and proteins have been shown to be involved in the metastatic development process, from the primary to the metastasis sites. Moreover, recent studies have focused on creating highly sensitive and specific microarrays to predict patient prognosis [6,7,8]. For example, studies have targeted epithelial cell adhesion molecule (*EpCAM*) [9], forkhead box M1 (*FOXM1*) [8], cyclin-dependent kinase 1 (*Cdk1*), tumor suppressor p53-binding protein 1 (*TP53BP1*), and cyclin-dependent kinase inhibitor 1B (*CDKN1B*) [6]. 

Leucine-rich repeat-containing G-protein-coupled receptor 5 (*LGR-5*) is a new target gene that activates the *Wnt* signaling pathway, facilitates tumor development and tumor cell signal transduction, and is reported in several malignancies such as colorectal cancer [10], ovarian tumors [11], basal cell carcinoma [12], breast cancer [13], and esophageal adenocarcinoma [14]. However, only a few studies have focused on the relationship between *LGR-5* expression and HCC prognosis, particularly in HBV and HCV infection groups. Therefore, in this study, we investigated the role of *LGR-5* expression in HCC by comparing tumor and normal tissue specimens from Taiwanese people using immunohistochemistry (IHC) staining to determine if *LGR-5* expression was an independent clinical outcome indicator of HCC.

## 2. Patients and Methods

### 2.1. Patients 

This prospective study included HCC patients admitted to the Division of General Surgery, Department of Surgery, Changhua Christian Hospital, Taiwan between November 2013 and September 2017. The inclusion criteria included newly diagnosed HCC patients, while the exclusion criteria included parameters, such as age <18 years, pregnancy, and treatments involving chemotherapy, targeted therapy, or surgical intervention. After selection, the patients underwent curative surgical intervention. The detailed demographic data, clinical data, pathological stage, and surgical outcomes were recorded. After tumor resection, the tumor tissues and matched adjacent normal liver tissues were investigated via immunohistochemical staining. A research team performed follow-ups and recorded the death, censorship, or lack of follow-up. Follow-ups were performed from the date of surgical intervention to a patient’s last visit or death. This study was approved by the Institutional Review Board of Changhua Christian Hospital (CCH IRB number: 170909). Informed consent was obtained from all HCC patients. 

### 2.2. IHC and Scoring

The anti-LGR5 antibody (orb137136) was purchased from Biorbyt (Cambridge, UK) and the anti-β-catenin antibody (610154) was purchased from BD Transduction Laboratories™ (Franklin Lakes, NJ, USA). The specimens were embedded in paraffin, cut into 4 μm-thick sections, attached to slides, and coated with poly-L-lysine. After deparaffinizing and rinsing with 10 mM Tris-HCl (pH 7.4) and 150 mM sodium chloride, the slides were treated with methanol and 3% hydrogen peroxide, and then placed in a 100 °C heating chamber for 20 min in 10 mM citrate buffer. Next, they were incubated with LGR-5 (1:100) and β-catenin (1:300) antibody solutions for 1 h. The slides were then washed with phosphate-buffered saline and LGR-5 antibodies were detected using the EnVision Detection Systems, Peroxidase/DAB, Rabbit/Mouse kit (Dako, Glostrup, Denmark). Finally, the slides were analyzed under a microscope (BX50, OLYMPUS, Japan). The negative samples and the control group were processed without the primary antibody. LGR-5 expression results were evaluated by professional pathologists and the scoring system was defined by two aspects: staining intensity and percentage of positive cells. The staining intensity was categorized into the following 4 grades [15,16]: 0, no expression; 1, weak expression; 2, moderate expression; and 3, strong expression. The IHC score was calculated using the following formula: staining intensity × percentage of positively labeled cells. The score ranged from 0 to 300.

### 2.3. Statistical Analysis

The correlation results between the clinicopathological parameters and the LGR-5 expression were investigated by the chi-square analysis and paired-sample *t*-test using the SPSS software (Version 13.0; SPSS Inc., Chicago, IL, USA). The results of the variables and survival data were analyzed by the log-rank test. Survival curves were plotted by the Kaplan–Meier method, and the Cox’s proportional hazards regression model was used for assessing the relationship between the variables and survival data. Statistical significance was defined as a *p*-value < 0.05.

## 3. Results

### 3.1. Patient Characteristics

In total, this study included 352 patients aged 14–89 years, with a mean age of 63.0 ± 11.9 years (standard deviation). Of these, 282 (80.1%) were male and 70 (19.9%) were female patients. Majority of the patients had a Child–Pugh score A (Child–Pugh points: 5.23 ± 0.73) and were in clinical stage I and II (78.4%), with a mean tumor size of 54.4 ± 46.0 mm. The prevalence rates of hepatitis B and C were 52.6% and 33.2%, respectively. After tumor excision, the pathological reports revealed poor differentiation in 184 patients (52.3%), followed by moderate differentiation in several patients (164 patients, 46.6%), and remarkable differentiation in 4 patients (1.1%). The follow-up duration for the HCC patients was 809.1 ± 418.7 days. During this period, 61 (17.3%) patients revealed tumor recurrence history. The characteristics of all HCC patients are listed in Table 1.

### 3.2. LGF-5 Expression 

**Figure 1:**
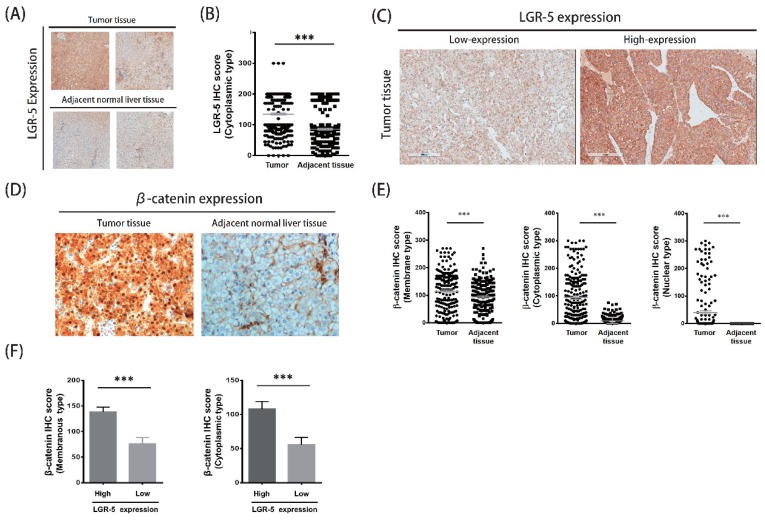
The LGR-5 expression and co-staining β-catenin protein in tumor and matched adjacent normal liver tissue of HCC patients. (**A**) The expression of LGR-5 in tumor and adjacent normal liver tissue. (**B**) The levels of LGR-5 expression in tumor and non-tumor liver tissues of HCC patients. (**C**) The high expression and low expression of tumor tissue. (**D**)The co-staining of β-catenin expression in tumor and matched adjacent normal liver tissue of HCC patients. (**E**) Three types of β-catenin expression were higher in tumor and non-tumor liver tissues of HCC patients. (**F**) The β-catenin expression was associated with LGR-5 expression.

In IHC staining, the results of LGR-5 expression in both HCC tissues and matched adjacent normal liver tissues are presented in Figure 1. The LGR-5 expression was higher in the tumor tissues than in the normal liver tissues (Figure 1A). The IHC score of LGR-5 level in the HCC tissues was significantly higher than that of the paired adjacent normal liver tissues (median IHC score: 134.95 vs. 85.50, *p* < 0.001; Figure 1B). According to the LGR-5 expression level, all patients with median IHC score of LGR-5 in the tumor group (cutoff value: 135) were further categorized into two groups: low expression and high expression (Figure 1C). The co-staining of the LGR-5 downstream protein, β-catenin, had similar results. The expression of β-catenin in HCC tissues was significantly higher than the matched adjacent normal liver tissues (Figure 1D). Three types of β-catenin expression were isolated, including membrane type, cytoplasmic type, and nuclear type, and also presented higher β-catenin expression in tumor tissue (Figure 1E). The β-catenin expression was associated with LGR-5 expression (Figure 1F).

We promoted the hypotheses that the high-expression LGR-5 group would have a poorer clinical outcome than the low-expression group. Among the clinicopathological parameters, the differentiation type and survival days were positively correlated with high LGR-5 expression (*p* < 0.05; Table 1). The correlation between high LGR-5 expression and HBV or HCV was not significant (*p* = 1.000, 0.308). The survival analysis results in the HCC patients revealed a significant difference in the three factors, namely recurrence, clinical stage, and LGR-5 expression (*p* < 0.05; Table 2). The median survival time was 804 and 599 days in the nonrecurrent and recurrent groups, respectively (*p* < 0.001). In clinical stage, the patients with stage I and II had 818 median survival days and those with stage III or IV had 531 days (*p* < 0.001), whereas, the median survival time in the high and low LGR-5 expression groups was 741 days (survival rate, 80.9%) and 755 days (survival rate, 89.6%), respectively. The correlation of other clinical outcomes is listed in Table 2. 

**Figure 2:**
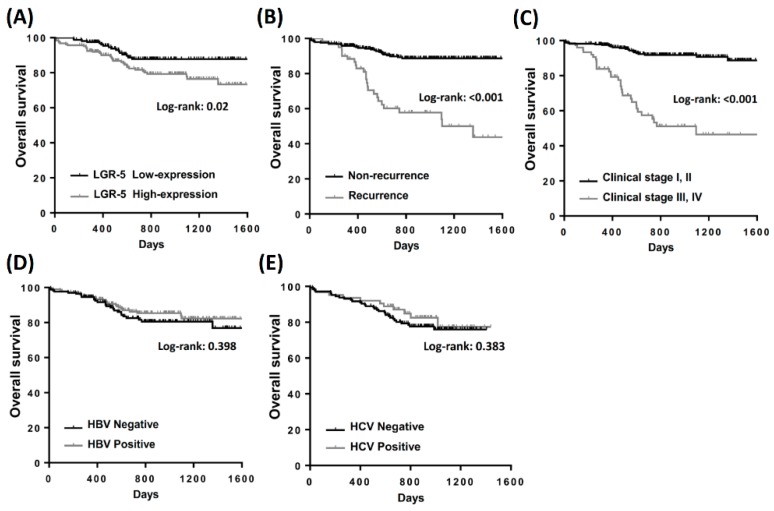
Kaplan–Meier analysis of the influence of (**A**) LGR-5 expression, (**B**) recurrence, (**C**) clinical stage, (**D**) hepatitis B virus infection, and (**E**) hepatitis C virus infection in total patients with HCC.

**Figure 3:**
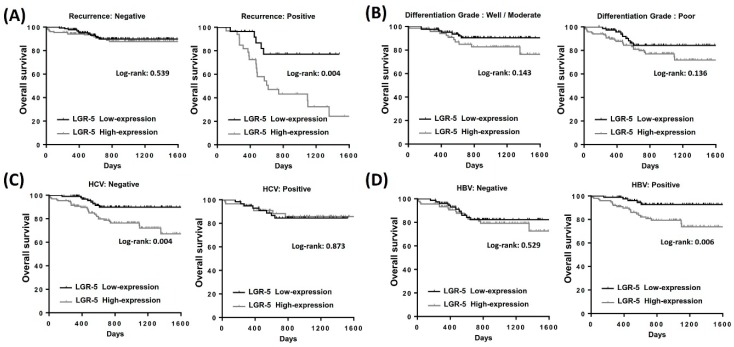
Kaplan–Meier analysis of LGR-5 expression in subgroup analysis in total patients with HCC. (**A**) The overall survival rate in recurrence group and non-recurrence group. (**B**) The overall survival rate in well/moderate differentiation grade group and poor differentiation grade group. (**C**) The overall survival rate in hepatitis C virus infection group and non-hepatitis-C-virus-infected group. (**D**) The overall survival rate in hepatitis B virus infection group and non-hepatitis-B-virus-infected group.

**Figure 4:**
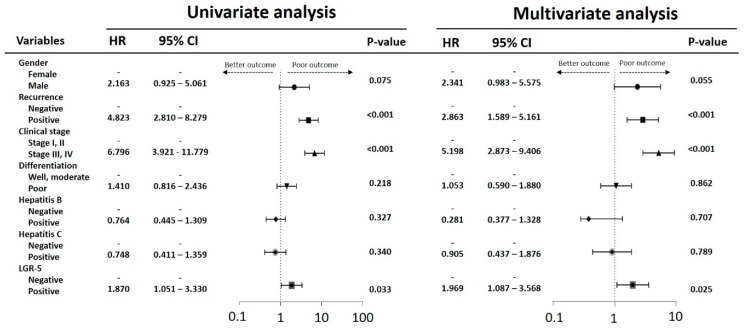
Univariate and multivariate analysis of influence of clinical characteristics in HCC patients.

Overall survival analysis indicated that patients with a recurrent history, high clinical stage, poor differentiation, and high LGR-5 expression had low survival rates. The Kaplan–Meier analysis revealed that high expression of LGR-5 had fewer overall survival days than those with low LGR-5 expression (Figure 2A). Similar results were observed during the recurrent history and high clinical stage in patients with HCC (Figure 2B,C). These results suggest that high LGR-5 expression is crucial for the clinical outcomes in patients with HCC. The survival analysis in the HBV or HCV groups was insignificant and no significant different trend of overall survival outcome (Figure 2D,E). In the subgroup analysis, high expression of LGR-5 impaired the outcome in the recurrent group (Figure 3A). High expression of LGR-5 in both differentiation grades indicated a poor clinical outcome (Figure 3B), and significantly impaired the hepatitis B positive and hepatitis C negative groups (Figure 3C,D). Cox regression analysis confirmed a prognostic significance of a recurrent history, high clinical stage, and high LGR-5 expression (Figure 4).

## 4. Discussion

LGR-5, also known as G-protein-coupled receptor 49 or 67 (GPR49 or 67), is a *Wnt* signaling pathway target gene that activates R-spondin 1 (*RSPO1*) and *Wnt-3A*, and cointernalizes with LDL receptor related protein 6 (*LRP6*) and Frizzled-5 (*FZD5*). It is expressed in several tissues and organs, including the muscles, spinal cord, and brain, and is particularly expressed in stem cells, thus serving as a novel biomarker. *LGR-5* is crucial in embryogenesis, tumor development, and tumor cell signal transduction. In recent years, several reports revealed that *LGR-5* overexpression was observed in various tumors, such as colorectal tumors [10], ovarian tumors [11], basal cell carcinoma [12], and esophageal adenocarcinoma [14]. However, studies on LGR-5 expression in human hepatic carcinoma are scarce. In 2007, Cao et al. [17] reported a new concept that suggested LGR-5 expression promoted cancer stem cell traits and chemoresistance in cervical cancer. Interestingly, LGR-5 appears to not only be a bona fide marker, but also a tumor promoter in cervical cancer via the Wnt/β-catenin pathway. Here, we build on the hypothesis that suggests *LGR-5* overexpression to be a good marker for HCC prognosis.

Similar to the reports of Liu et al. [18], LGR-5 expression was found to be significantly higher in liver tumor tissues than in the adjacent non-tumor tissues. In our study, three major factors were associated with clinical outcomes: LGR-5 expression, clinical stage, and recurrent history. In the clinical outcome analysis, *LGR-5* overexpression revealed poor clinical outcome with significantly shorter survival days and poor survival rates. A similar result was reported Liu et al. [18], which showed that upregulated LGR-5 correlated with high clinical stage, recurrence, and metastasis. These findings supported our results, which indicated that upregulated LGR5 correlated with poor clinical outcomes as seen in the Kaplan–Meier analysis. In 2017, Chen et al. [19] studied a small sample size of HCC patients (*N* = 66) when investigating the role of LGR-5. Although the report revealed the correlations between LGR-5 expression, clinical stage and overall survival days, no difference in the tumor size, HCV, or Child–Pugh scores was detected between overexpressed and underexpressed LGR-5. Interestingly, no significant difference was also observed between HCV, HBV, and LGR-5 expression in both studies. To clarify this point, a subgroup analysis was carried out, which indicated that HCV-negative patients with high LGR-5 expression had poor clinical outcomes when compared to HCV-positive patients (*p* = 0.004). In HBV-positive patients, LGR-5 overexpression promoted lower overall survival (*p* = 0.006), presented a poor outcome in the differentiation grade using histology differential grading and indicated a significantly poor clinical outcome in recurrent patients (*p* = 0.004). Moreover, Kaplan–Meier analysis revealed that LGR-5 expression was crucial in patients with recurrent histories, HCV-negative groups, and HBV-positive groups.

In Taiwan, the prevalence rate of HBV and HCV is high. These viruses may cause local inflammation and promote inflammasome formation, eventually causing hepatocarcinogenesis. Recently, several oncogenes and tumor suppressor genes, such as EpCAM, Wnt, β-catenin, and p53, were investigated to understand the correlation between hepatocarcinogenesis and hepatitis B and C; however, only a few studies focused on the new biomarker, LGR-5. Interestingly, this study found that the correlation between LGR-5 overexpression and recurrent histories in HCV-negative and HBV-positive groups probably induced poor outcomes. Furthermore, we hypothesize that HBV may trigger LGR-5 activity, eventually leading to hepatocarcinogenesis, and that HCV infection may impair the function of LGR-5. However, the detailed mechanism of the interaction between HBV/HCV and LGR-5 overexpression remains unclear, and requires further basic and clinical trials to confirm these speculations. Despite our important findings, the limitations of this study include its small sample size (*n* = 352) and limited patient numbers in advanced clinical stage III/IV. Although these limitations may have influenced our clinical results, LGR-5 expression was still shown to be a significant prognostic factor. Thus, we speculate that LGR-5 overexpression is associated with poor prognosis in HCC, particularly in recurrent histories, HCV-negative groups and HBV-positive groups. 

## 5. Conclusions

In conclusion, this study revealed an essential role of LGR-5 in HCC patients. LGR-5 is an important risk factor for poor survival and is suggested for use in routine IHC staining. Further studies are required to analyze LGR-5 expression in selective chemotherapy regimens, radiotherapy, and immunotherapy after surgical intervention. Our preliminary data revealed that LGR-5 expression in HCC might be a novel target in recurrent history, in the HCV negative and HBV positive groups. Thus, developing anti-LGR-5 therapy is necessary for HCC and should be explored in the future.

## Figures and Tables

**Table 1:** Relationship of clinicopathological characteristics with LGR-5 expression in hepatocellular carcinoma (HCC) patients.

Variables	Total	LGR-5		*p*-Value
		Low Expression	High Expression	
Age (years)	63.0 ± 11.9	63.3 ± 12.2	62.6 ± 11.6	0.935
Gender, n				
Female	70	35	35	0.506
Male	282	128	154	
Recurrence, n				
Negative	291	135	156	1.000
Positive	61	28	33	
Differentiation, n				
Well/ Moderate	168	92	76	0.003
Poor	184	71	113	
Clinical stage, n				
Stage I, II	276	127	149	0.897
Stage III, IV	76	36	40	
Tumor size, (mm)	54.4 ± 46.0	56.5 ± 47.7	52.6 ± 44.7	0.319
Child–Pugh score	5.23 ± 0.73	5.21 ± 0.65	5.24 ± 0.78	0.545
Survival days	809.1 ± 418.7	800.1 ± 396.7	816.8 ± 437.9	0.029
Survive, n	298	146	152	0.018
Hepatitis B, n				
Negative	167	77	90	1.000
Positive	185	86	99	
Hepatitis C, n				
Negative	235	104	131	0.308
Positive	117	59	58	

**Table 2:** Univariate analysis of overall survival in total HCC patients.

Variables	Number	Overall Survival		Log-Rank
		Median Survival Days	Survival (%)	
Gender				
Female	70	845	91.4	0.068
Male	282	741	83.3	
Recurrence				
Negative	291	804	90.3	<0.001
Positive	61	599	59.0	
Differentiation				
Well/Moderate	168	804	86.9	0.215
Poor	184	736	83.1	
Clinical stage				
Stage I, II	276	818	92.0	<0.001
Stage III, IV	76	531	58.7	
Hepatitis B				
Negative	167	812	82.1	0.607
Positive	185	740	83.3	
Hepatitis C				
Negative	235	732	82.1	0.463
Positive	117	848	84.5	
LGR-5				
Low	163	755	89.6	0.020
High	189	741	80.9	
